# Stem cell therapy targets the neointimal smooth muscle cells in experimentally induced atherosclerosis: involvement of intracellular adhesion molecule (ICAM) and vascular cell adhesion molecule (VCAM)

**DOI:** 10.1590/1414-431X2020e10807

**Published:** 2021-05-24

**Authors:** R.M. Hashem, L.A. Rashed, R.M. Abdelkader, K.S. Hashem

**Affiliations:** 1 Department of Biochemistry, Faculty of Pharmacy, Beni-Suef University, Beni-Suef, Egypt; 2 Department of Biochemistry, Faculty of Medicine, Cairo University, Cairo, Egypt; 3 Department of Biochemistry, Faculty of Veterinary Medicine, Beni-Suef University, Beni-Suef, Egypt

**Keywords:** Atherosclerosis, Intracellular adhesion molecule (ICAM), Vascular cell adhesion molecule (VCAM), Smooth muscle cells, Stem cells

## Abstract

Smooth muscle cells (SMCs) are currently considered a central pivotal player in pathogenesis and development of atherosclerotic lesions. As consequence of vascular injury, SMCs migrate from the tunica media into the tunica intima layers where they contribute to neointimal formation by converting into foam cells and producing pro-inflammatory and oxidative stress markers. We targeted the replacement of neointimal SMCs by using the mesenchymal stem cells (MSCs) therapy in experimentally induced atherosclerosis in an attempt to improve the atherosclerotic lesion and its concomitant complications. Rats were divided into 4 groups (n=20). Control group: rats kept on a standard chow diet; atherosclerotic group: rats received the atherogenic diet; stem cells-treated group: rats were injected with CD34^+^ stem cells (6×10^6^ cells in 0.5 mL PBS in rat tail vein) and maintained on the atherogenic diet; and resveratrol-treated group: rats were supplemented orally with resveratrol at a dose level 3 mg/kg per day and the atherogenic diet. After 12 weeks, rats were euthanized, blood samples were collected for separation of serum, and abdominal aortas were excised for further biochemical, molecular, and histopathological investigations. We used resveratrol, the well-established anti-atherosclerotic drug, as a benchmark to assess the efficacy of stem cell therapy. MSCs treatment revealed significant amelioration in both histopathological and biochemical patterns as evidenced by decreased foam cells formation, ICAM-1, VCAM, M-CSF, iNOS, COX-2, and TNF-α. We concluded that MSCs therapy significantly replaced the neointimal SMCs and decreased adhesion molecules as well as the oxidative and inflammatory markers in atherosclerosis.

## Introduction

There is growing evidence that stem cells and smooth muscle progenitor cells contribute to arteriosclerosis by differentiating into smooth muscle cells (SMCs) in the intima layer (1). This means that stem/progenitor cells may play an important role in the pathogenesis of atherosclerosis. Recently, accumulating evidence indicates that mechanical forces and cytokines can influence stem cell differentiation into vascular SMCs (1).

SMCs are the main cell type in early arterial intimal thickening and a major component of most stages of human atherosclerosis (2). Intimal SMCs have monocyte-macrophage characteristics such as the expression of c-fms and scavenger receptor gene (3).

Like monocyte-derived macrophages, SMCs express scavenger receptors and become foam cells on exposure to lipoproteins (4). Allahverdian et al. present data suggesting that a large proportion (at least 50%) of total foam cells in human coronary intima are derived from SMCs rather than from monocytes (5). Furthermore, various cytokines such as tumor necrosis factor α (TNF-α) and macrophage-colony stimulating factor 1 (M-CSF 1) are reported to be synthesized and secreted by vascular cell components including monocyte-macrophages and SMCs. M-CSF, an 85-kDa homodimeric glycoprotein, specifically promotes growth and differentiation of the monocyte macrophage lineage and activates various functions of mature macrophages. The effects of M-CSF are mediated through binding to specific, high-affinity surface receptors encoded by the c-fms proto-oncogene. Interestingly, c-fms was found in SMCs isolated from experimental arteriosclerosis, but not in the normal medial SMCs (6). The stem cell has the ability to differentiate into a variety of cells to replace dead cells or to repair tissue (7).

The inducible isoform nitric oxide synthase (iNOS) is normally absent in the vasculature under physiological conditions. Its expression is induced in blood vessels in pathological situations, such as inflammation, sepsis, or oxidative stress (8). It is reported that over-expression of iNOS is associated with atherosclerotic lesions as it promotes the formation and activity of peroxynitrite. This may be important in the pathology of atherosclerosis, which contributes to lipid peroxidation and to vascular damage (9).

Over-expression of reactive oxygen species (ROS) decreases antioxidant pathways, ultimately leading to production of pro-oxidant species. These modifications may also negatively influence platelet and clotting activation, eventually leading to cardiovascular complications. The cardiovascular risk factors that are associated with atherosclerosis such as hypercholesterolemia, hypertension, diabetes mellitus, and smoking enhance ROS generation. Key molecular events in atherogenesis such as oxidative modification of lipoproteins and phospholipids, endothelial cell activation, and macrophage infiltration/activation are facilitated by vascular oxidative stress (10).

COX-2 is an inducible form of cyclooxygenase enzyme and it can be found in most tissues under normal physiological conditions. The over-expression of COX-2 could be attributed to many factors such as increased free radicals and cytokines. COX-2 is powerfully implicated in the development of atherosclerosis (11)

To be effective in suppression, slowing of progression, and regression of atherosclerosis, a drug should have inhibitory effects on the numerous players cited above. Therefore, we tested the effect of injecting stem cells into hypercholesterolemic rats to pinpoint the efficacy of replacement of SMCs in atherosclerotic lesions with stem cells, and to address the consequent effects on the potential players in the mechanisms of atherosclerosis including foam cells, adhesion molecules [vascular cell adhesion molecule (VCAM-1) and intracellular adhesion molecule (ICAM-1)], chemokines such as M-CSF-1, inducible enzymes such as iNOS and COX-2, and oxidative stress. Additionally, we estimated the efficiency of using stem cell therapy compared to one of the well-documented natural antiatherosclerotic drugs, resveratrol.

Resveratrol is a polyphenolic phytochemical that is biosynthesized by certain edible plants such as grape, peanut, and berry in response to phytogenic insults or pathogens. Resveratrol supplementation in a hypercholesterolemic model ameliorated atherosclerotic plaque and decreased the intimal layer thickness (12). It also elicited antiatherogenic properties in apoE-deficient mice. The mechanisms for resveratrol-mediated antiatherogenic effects include the reduction of M-CSF and the down-regulation of ICAM-1 and of VCAM-1 in the atherosclerotic vessels (13).

Hence, the present study was mainly aimed to shed light on the effect of stem cell therapy and the efficacy of stem cell replacement by proliferating SMCs in hypercholesterolemic atherosclerotic aortic lesions in rats as well as to compare the potential of stem cell therapy with the well-documented anti-atherosclerotic drug resveratrol.

## Material and Methods

### Animals

Eighty male Albino Wistar rats (200±25 g), purchased from the Animal House of the Research Institute of Ophthalmology (Egypt), were kept in controlled conditions of temperature (25±3°C) with a 12-h light/dark cycle. Rats had free access to water and a standard laboratory chow diet. The protocol of the current study and all procedures for agent administration and tissue collection were in accordance with the National Institutes of Health (NIH, USA) guide for the care and use of laboratory animals (NIH Publications No. 8023, revised 1978, No. 020-119). Body weight was recorded weekly.

### Isolation, propagation, identification, and labeling of bone marrow-derived MSCs from rats

Bone marrow was isolated from the tibiae and femurs of 6-8 white albino male rats and cultured in Dulbecco's modified Eagle's medium (DMEM, Gibco/BRL, USA) and 10% FBS. The density gradient (Ficoll/Paque, Pharmacia, Canada) was used to isolate nucleated cells, which were resuspended in DMEM supplemented with 10% FBS and 1% penicillin/streptomycin (Gibco/BRL) at 37°C and 5% CO_2_/95% humidified air. At 80% confluence, the cells were washed with phosphate buffered saline (PBS) and then trypsinized with 0.25% trypsin-1 mM EDTA for 5 min at 37°C and resuspended in growth medium containing serum (14). Two weeks later, the confluent cells were trypsinized and counted.

Cells were identified as being MSCs by their ability to differentiate into osteocytes (15), chondrocytes, and adipocytes (16). Cells were seeded in an osteocytogenic differentiation medium (DMEM, 10% FBS, 100 nM dexamethasone, 0.25 mM ascorbic acid, and 10 mM β-glycerol phosphate), and confirmed by Alzarin red staining. Differentiation of MSCs into chondrocytes was carried out by adding 500 ng/mL BMP-2 (R&D Systems, USA) and 10 ng/mL TGF-b3 (Peprotech, UK) (17), and confirmed by morphological changes and Alcian blue staining. Differentiation of MSCs into osteocytes was achieved by growing cells in a media containing 0.5 µM 3-isobutyl-1-methylxanthine, 200 µM indomethacin, 10 µM insulin, and 1 µM DEX. In addition to morphological changes, differentiation of MSCs into adipocytes was confirmed by oil-red staining. MSCs were also identified by surface markers CD29 (+ve), CD34 (-ve), CD90 (+ve), and CD105 (+ve) using flow cytometry.

Cells were labeled with PKH26 fluorescent linker dye (Sigma-Aldrich, USA). PKH26 is a red fluorochrome (excitation 551 nm and emission 567 nm). The linkers are physiologically stable and have no toxic side-effects on the cells. Cells were centrifuged at 800 *g* for 10 min at 25°C, washed, pelleted, and suspended in dye solution, and then injected intravenously into rat tail vein. One month later, aortic arteries were harvested and examined with a fluorescence microscope (Leica, Germany) to detect and trace the cells (18). Rats were injected with CD34^+^ stem cells (6×10^6^ cells in 0.5 mL PBS in rat tail vein).

### Experimental design

One week after acclimatization, twenty rats were kept on the standard chow diet as a control group while the others were switched to the atherogenic diet, which consisted of 10% soybean oil, 1.5% cholesterol, and 1.8 million units of vitamin D/kg for 6 weeks (19). Rats that achieved a plasma cholesterol level >200 mg/dL were selected for this study. The atherosclerotic rats were randomly divided into three experimental groups of 20 rats each. The atherosclerotic group received the atherogenic diet, the stem cells-treated group was injected with CD34+ stem cells [6×10^6^ cells in 0.5 mL PBS in rat tail vein (20)] and maintained on the atherogenic diet, and the resveratrol-treated group was supplemented orally with resveratrol (Sigma-Aldrich, Egypt) by oral gavage in a dose of 3 mg/kg body weight per day and maintained on the atherogenic diet; the selected dose was in accordance with Wang et al. (12). Stock solution of resveratrol was prepared in distilled water. Each rat received 0.27±0.03 mL of stock solution according to the body weight. After 12 weeks, the rats were euthanized and blood samples were collected for separation of serum, which was kept at −20°C for further biochemical investigation. Abdominal aortas were excised, cleaned of adherent fat and connective tissue, and cut into ring segments. Aortas were divided into three parts: the first part was stored at −80°C for RT-PCR; the second part of the aortas was preserved in 10% formalin solution for histopathological examination; and the third part of the aortas was homogenized in 5 mL PBS using a homogenizer (Ortoalresa, Spain) and centrifuged at 10,000 *g* for 15 min at 4°C. The supernatants were collected and used directly for measurement of levels of the oxidative stress-related parameters.

### Biochemical analysis

Enzymatic methods using commercially available kits (Spinreact, Spain) were used for colorimetric determination of serum levels of total cholesterol, total lipid, low-density lipoprotein cholesterol (LDL-C), high-density lipoprotein cholesterol (HDL-C), and triglycerides. Assay kits for determination of glutathione (GSH), catalase, total antioxidant capacity (TAC), and malondialdehyde (MDA) were purchased from Bio Diagnostic (Egypt). Percentage of DNA fragmentation assay kit was purchased from Fisher Scientific Company (USA).

### Detection of M-CSF, ICAM, and VCAM by ELISA

Serum concentrations of M-CSF, ICAM, and VCAM were measured using commercial enzyme immunoassay kits (ELISA, R&D Systems, UK), according to manufacturer instructions.

### Detection of COX-2, iNOS, and TNF-α gene expression

RNA extraction and RT-PCR were performed as described previously (21). Briefly, total RNA was extracted from aorta tissue homogenates using RNeasy Purification Reagent (Qiagen, USA). For cDNA preparation, 5 µg total RNA was reverse-transcribed with oligonucleotide (dT) 18 primer and denatured at 70°C for 2 min. The reaction mixture consisted of 50 mM KCl, 50 mM Tris HCl (pH 8.3), 0.5 mM dNTP, 3 mM MgCl_2_, 1 U/mL RNase inhibitor, and 200 units of Moloney murine leukemia virus reverse transcriptase at 42°C for 1 h, and then stopped at 92°C. RT-PCR amplification was conducted using 25 μL amplification mixtures containing SYBR Green PCR Master Mix (Applied Biosystems, USA) and 200 ng of each primer (Table 1). Data were analyzed with the ABI Prism 7500 sequence detection system software and quantified using the Sequence Detection Software v1.7 from PE Biosystems (USA). Relative expression was calculated using the comparative threshold cycle method. All values were normalized to the beta actin gene (22), as described by Livak et al. (23).

**Table 1:** Sequences of forward and reverse primers.

COX-2	forward: 5′-ACACTCTATCACTGGCATCC-3′reverse: 5′- GAAGGGACACCCTTTCACAT-3′	Gene ID: 7751820Accession number on the chromosome: NC_012389.1
iNOS	forward: 5′-CGGTTCACAGTCTTGGTGAAAG-3′	Gene ID: 24599
	reverse: 5′-CAGGTGTTCCCCAGGTAGGTAG-3′	Accession number on chromosome: NC_005109.4
TNF-α	forward: 5′-CGTCGTAGCAAACCACCAAG-3′	Gene ID: 24835
	reverse: 5′-ACACAGAGCAATGACTCCAAAG-3′	Accession number on chromosome: NC_005119.4
β-actin	forward: 5′-ATCATGTTTGAGACCTTCAACACC-3′	Gene ID: 11461
	reverse: 5′-TAGCTCTTCTCCAGGGAGG-3′	Accession number of chromosome: NC_000071.6

### Histological examination

Aorta tissues were fixed in 10% formalin for 48 h, washed and dehydrated in graduated ethyl alcohol, cleared in xylene, and embedded in paraffin wax blocks. Sections 5-μm thick were stained with hematoxylin and eosin (H&E) and examined under a light microscope (Leica) for determination of pathological changes.

### Statistical analysis

Statistical analysis was carried out using GraphPad Instate software (version 3, ISS, Italy), and one-way analysis of variance followed by the Tukey-Kramer multiple comparison *post hoc* test were used to establish significant differences between groups.

## Results

### Characterization and homing of BM-MSCs in aortic tissue

**Figure 1:**
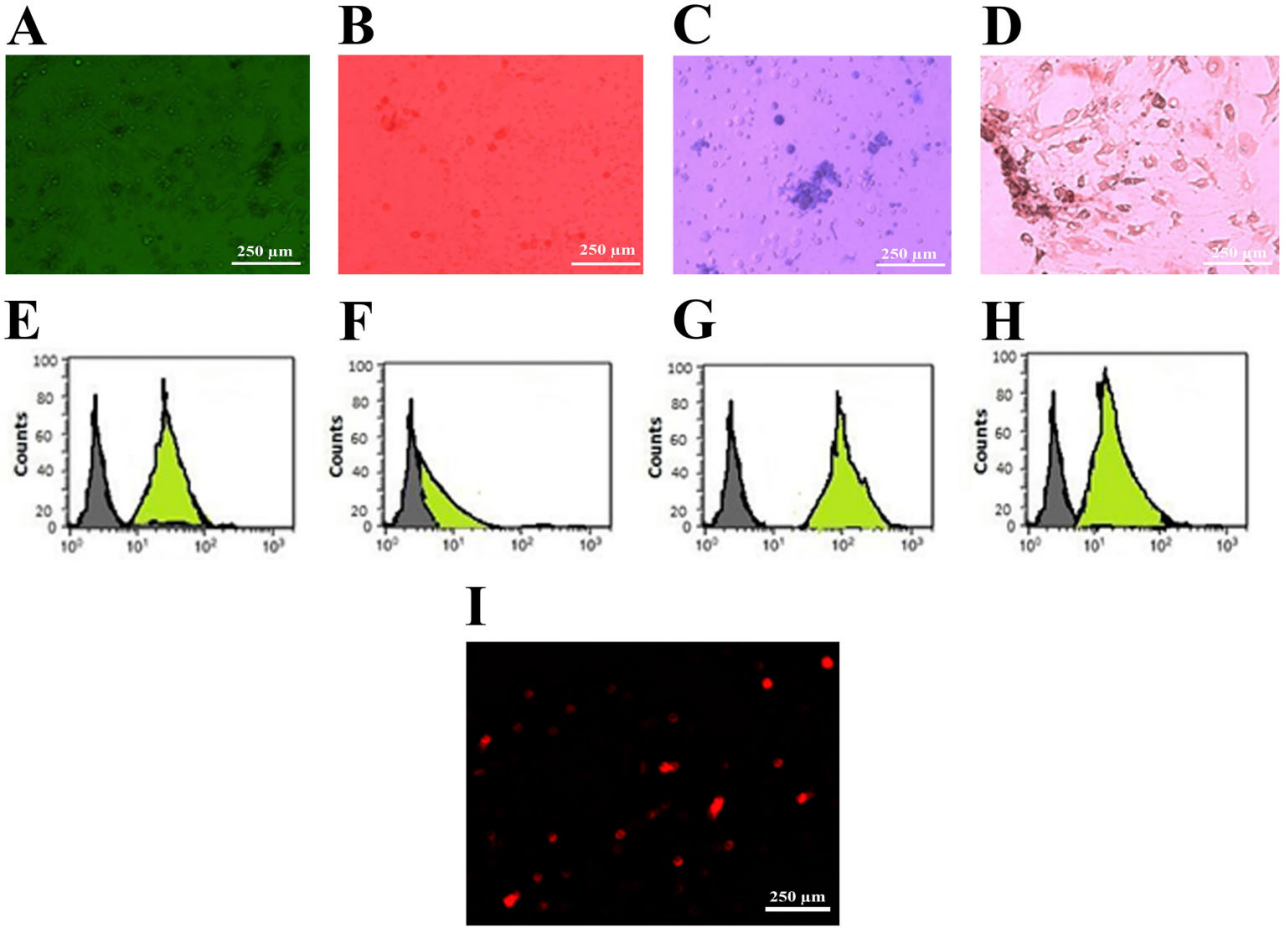
Characterization and homing of bone marrow smooth muscle cells (BM-MSCs) in aortic tissue of rats experimentally induced with atherosclerosis. Morphological and histological staining of differentiated BM-MSCs into undifferentiated MSCs (**A**), and MSCs differentiated into osteoblasts stained with Alizarin red stain (**B**), chondrocytes stained with Alcian blue stain, and (**C**), adipocytes stained with oil red stain (**D**). Flow cytometry charts for cells CD29+ve (**E**), CD34-ve (**F**), CD90 +ve (**G**), and CD105 +ve (**H**). **I**, Photomicrograph of a section of aorta from atherosclerotic group treated with stem cells showing positive immunofluorescent. Scale bar 250 μm.

Bone marrow (BM)-MSCs were identified by their adhesive ability and fusiform shape (Figure 1A) as well as their characteristic feature to differentiate *in vitro* into osteoblasts, chondrocytes, and adipocytes (Figure 1B-D). The immunophenotype of cells isolated from rat BM detected by flow cytometry were positive for CD29 (Figure 1E), CD90, and CD105 (Figure 1G and H) and negative for CD34-ve (Figure 1F). As illustrated in Figure 1I, BM-MSCs labeled with PKH26 fluorescent dye were detected in the aortic tissues confirming that these cells homed into the atherosclerotic aortic tissue of recipient rats one month after *iv* injection in their vein tails (Figure 1I).

### Detection of lipid profile

As shown in Table 2, administration of the diet enriched with cholesterol and vitamin D resulted in a significant (P<0.05) increment in the serum levels of total cholesterol (TC) (117%), triglycerides (TG) (100%), LDL-C (158%), and total lipids (216%). Although HDL-C was not significantly decreased compared to the normal group, the atherogenic index (TG/HDL-C) was increased two-fold. The serum levels of TC (32%), TG (33%), LDL-C (38%), and total lipids (28%) decreased in the resveratrol-treated group. The resveratrol-treated rats showed a significant decrease in TG/HDL-C (2.76 *vs* 4.1, P<0.05) compared to the atherosclerotic group. The stem cells-treated group did not show any hypolipidemic effect compared to the atherosclerotic rats.

**Table 2:** Total lipids, total acyl glycerol (TAG), low-density lipoprotein cholesterol (LDL-C), and high-density lipoprotein cholesterol (HDL-C) in of rats experimentally induced with atherosclerosis and treated with stem cells or resveratrol.

Groups	Cholesterol (mg/dL)	Total lipid (mg/dL)	LDL-C (mg/dL)	HDL-C (mg/dL)	TAG (mg/dL)	Atherogenic index (TAG/HDL)
Control	115.3±13.2	300.2±24.8	67.85±8.72	31.33±6.08	80.42±10.2	2.56
Atherosclerotic	250.8±23.6^a^	640.5±59.7^a^	175±15.6^a^	39±7.22^a^	160.0±17.2^a^	4.1^a^
Stem cells-treated	243.6±31.6^a^	630±72.4^a^	171.0±18.5^a^	38.6±7.32^a^	155.3±11.2^a^	4.08^a^
Resveratrol-treated	170.4±16.1^a,b^	460.5±38.9^a,b^	108.8±11.4^a,b^	38.8±7.51^a^	107.2±10.8^a,b^	2.76^b^

Data are reported as means±SE. ^a^P<0.05 compared to control group; ^b^P<0.05 compared to atherosclerotic group (ANOVA).

### Detection of oxidative stress

Experimentally-induced atherosclerosis resulted in oxidative stress as shown in Table 3. The serum levels of TAC, catalase, and GSH recorded a significant decrease (P<0.05) compared to the control rats while the lipid peroxidation marker MDA had a significant enhancement compared to the control rats. Stem cell administration increased levels of TAC, catalase, and GSH, and decreased levels of MDA compared with the atherosclerotic group (P<0.05). Resveratrol increased the levels of TAC, catalase, and GSH and downregulated the levels of MDA compared to the atherosclerotic group (P<0.05).

**Table 3:** Total antioxidant capacity (TAC), catalase, glutathione (GSH), and malondialdehyde (MDA) in rats experimentally induced with atherosclerosis and treated with stem cells or resveratrol.

Groups	GSH (mg/g tissue)	MDA (nmol/g tissue)	TAC (mM/L)	Catalase (U/g tissue)
Control	68.28±7.54	25.98±3.48	3.1±2.55	6.23±0.43
Atherosclerotic	37.53±2.95^a^	68.24±8.38^a^	1.45±0.13^a^	2.3±0.27^a^
Stem cells-treated	59.09±5.72^b^	32.96±2.81^b^	2.8±0.31^b^	5.55±0.47^b^
Resveratrol-treated	40.2±5.31^a,b^	43.57±3.68^a,b^	1.6±0.18^a,b^	3.15±0.26^a,b^

Data are reported as means±SE. ^a^P<0.05 compared to control group; ^b^P<0.05 compared to atherosclerotic group (ANOVA).

### Detection of M-CSF, ICAM, and VCAM by ELISA

**Figure 2:**
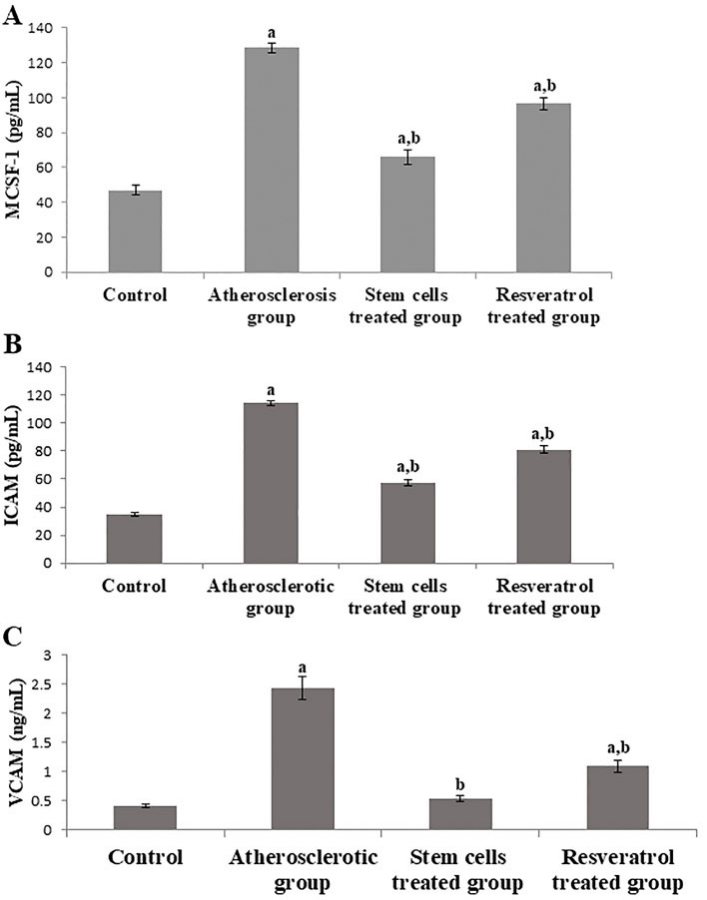
Protein levels of macrophage-colony stimulating factor 1 (MCSF-1) (**A**), intracellular adhesion molecule (ICAM) (**B**), and vascular cell adhesion molecule (VCAM) (**C**) in groups of rats experimentally induced with atherosclerosis, treated with stem cells or resveratrol, and control. Data are reported as means±SE. ^a^P<0.05 compared to control group; ^b^P<0.05 compared to atherosclerotic group (ANOVA).

Our results showed that the induction of atherosclerosis manifested a significant (P<0.05) increase in the levels of M-CSF and the adhesion molecules ICAM and VCAM compared to the control group. However, treatment with stem cells and resveratrol substantially reduced their high levels compared to the atherosclerotic group (P<0.05). Interestingly, stem cells-treated group revealed a more significant decrease in the levels of M-CSF and adhesion molecules than the resveratrol-treated one (P<0.05). There was no significant difference in protein level of VCAM between the stem cells-treated group and the control group (Figure 2 A-C).

### Detection of relative gene expression of COX-2, iNOS, and TNF-α

**Figure 3:**
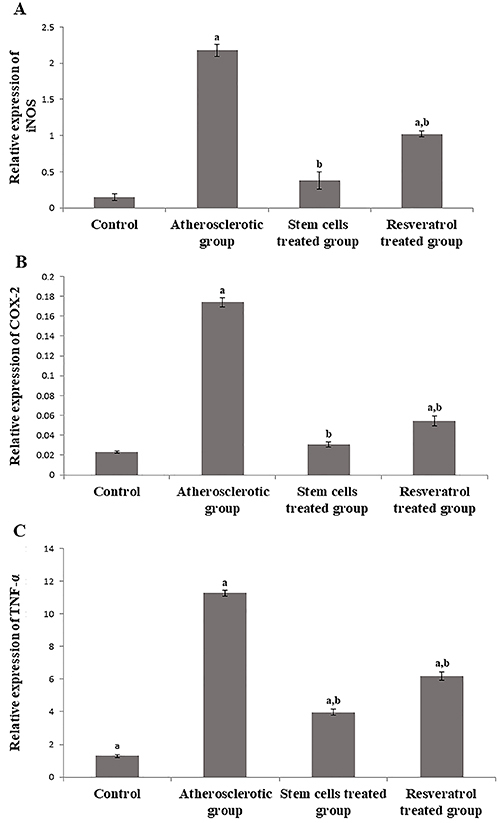
Inducible nitric oxide synthase (iNOS) (**A**), COX-2 (**B**), and tumor necrosis factor α (TNF-α) (**C**) in rats experimentally induced with atherosclerosis and treated with stem cells or resveratrol. Data are reported as means±SE. ^a^P<0.05 compared to control group; ^b^P<0.05 compared to atherosclerotic group (ANOVA).

As shown in Figure 3A-C, experimentally induced atherosclerosis caused a significant increase in the gene expression of COX-2, iNOS, and TNF-α compared to the control group, while stem cells treatment significantly reduced their levels compared to the atherosclerotic and resveratrol groups. The stem cells-treated group did not reveal the same statistical decrease in TNF-α levels compared with the control group. There was no significant difference in the gene expression of COX-2 and iNOS between the stem cells-treated group and the control rats.

### DNA fragmentation

**Figure 4:**
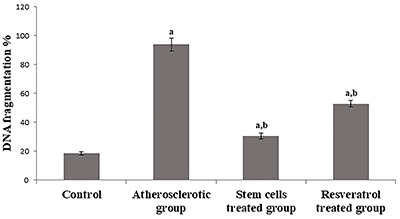
DNA fragmentation (%) in rats experimentally induced with atherosclerosis and treated with stem cells or resveratrol. Data are reported as means±SE. ^a^P<0.05 compared to control group; ^b^P<0.05 compared to atherosclerotic group (ANOVA).

The present study showed that the induction of atherosclerosis caused a significant increase of DNA fragmentation compared to the control group. Stem cells treatment significantly reduced the DNA fragmentation compared to the atherosclerotic and resveratrol groups (Figure 4).

### Histopathological findings

**Figure 5:**
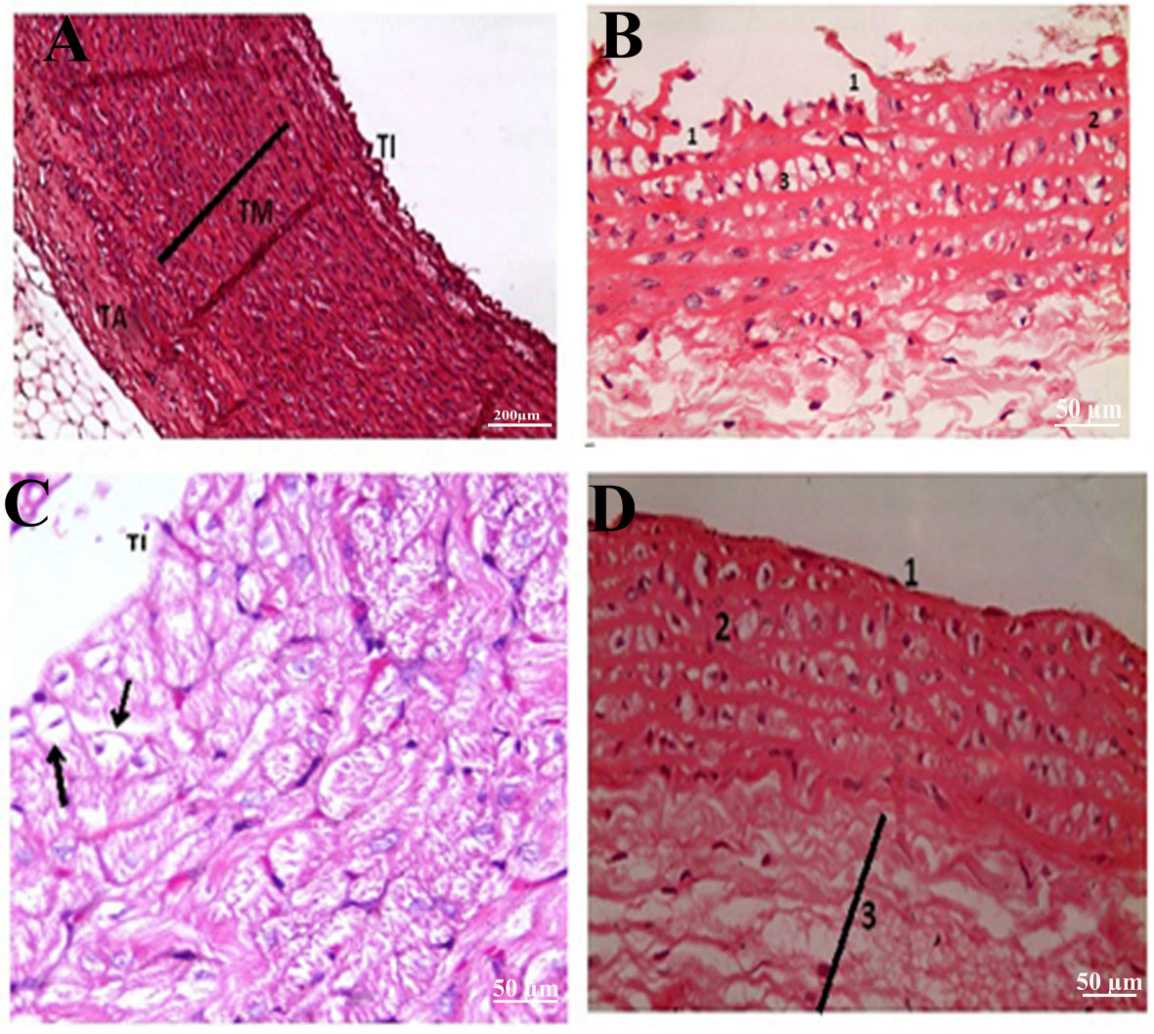
Histological changes of aortas of rats experimentally induced with atherosclerosis. **A**, Control group: three layers forming wall of aorta: tunica intima (TI), tunica media (TM) formed of concentrically-arranged smooth muscle, and tunica adventitia (TA) (H&E, ×200, scale bar 200 μm). **B**, Atherosclerotic group: desquamation and destruction of tunica intima with underlying lamina propria (1); tunica media shows a deposition of fat (2) with appearance of foam cells (3) (H&E, ×400, scale bar 50 μm). **C**, Stem cells-treated group: the slide shows normal appearance of the TI with no desquamation, TM appears with normal thickness with very little fat infiltration (head of black arrow) and no detected foam cells (H&E, ×400, scale bar 50 μm). **D**, Resveratrol-treated group: TI appears intact with no destruction or desquamation (1). Presence of foam cells in the TM (2) and increased thickness of the TA (3) (H&E ×400, scale bar 50 μm).

Histopathological examination (Figure 5) of sections of aortic tissues from the normal control group showed normal architecture, which consists of the three different layers, tunica intima (TI), tunica media (TM), and tunica adventitia (TA), which appeared normal (Figure 5A). Atherosclerotic aortic sections demonstrated substantial destruction in TI and appearance of foam cells in TM (Figure 5B). Sections from the stem cells-treated group showed restoration of these adverse effects, higher than the resveratrol-treated group, whereas desquamation and foam cells were significantly decreased in TI and TM, respectively, as illustrated in Figure 5C and D.

## Discussion

We tested the stem cell therapy efficacy in replacing the damaged cells in atherosclerotic lesions compared to the naturally extracted drug resveratrol that has established its anti-atherosclerotic effects in many previous studies (24). MSC express a range of chemokine receptors, allowing them to specifically migrate towards chemokine gradients. The migration of endogenous MSC is controversial. Strong evidence for the migration of MSC via the bloodstream is sparse. MSC are located in the bone marrow, from where they may migrate to other sites via mechanisms potentially similar to those exploited by hematopoietic stem cells. However, MSC may be resident in peripheral blood, which would make the specific identification of migrating MSC difficult. Nevertheless, the initial frequency of MSCs is considered to be low with generally less than 0.1% of BM mononuclear cells in a newborn and declining with age (25). MSC are relatively easy to isolate and expand in culture and, therefore, have potential as a therapeutic tool (26).

We induced an experimental atherosclerosis model as indicated histopathologically in Figure 5B that shows the characteristic foam cells and damaged endothelial cells. Also, we successively seeded MSCs labeled with PKH26 into the aortic tissue of atherosclerotic rats as confirmed by strong red autofluorescence. A widely accepted theory for the pathogenesis of arteriosclerosis is that proposed by Takahashi et al. (27), which states that one of the main contributors to the pathogenesis of arteriosclerosis are the SMCs. Furthermore, the theory suggests that as a consequence to vascular injury, SMCs migrate from the media into the intima, where they contribute to neointimal formation by converting into foam cells and producing extracellular matrix. As predicted, a significant increase in M-CSF in the atherosclerotic rats has been recorded. M-CSF is one of the crucial factors for monocyte/macrophage differentiation and proliferation, and for the survival of macrophages in the atherosclerotic lesions (27). Previous studies have reported that M-CSF receptor gene and scavenger receptor gene are induced on SMCs derived from the intima of atherosclerotic lesions (1).

The conversion of macrophages into foam cells is a critical step in the development of atherosclerosis and it runs through all stages of atherosclerosis (28). Zhang et al. (29) stated that administration of MSCs reduced the foam cell formation about 50% compared to the non-treated group. The ability of MSCs to reduce foam cells could be explained by the ability of MSCs to markedly inhibit the expression of certain receptors that are incorporated in the conversion of macrophages to foam cells such as CD36 and SRA1 while promoting the expression of ABCA1. Previous reports showed that MSCs administration reduced the development of inflammatory macrophage precursor cells. A potential influence of MSCs in control and treatment of atherosclerosis is the inhibition of both monocyte migration and local macrophage activation, proliferation, and differentiation in the pathogenesis of atherosclerosis (29).

Moreover, the stem cells-treated group showed a stronger decrease in the levels of M-CSF than the resveratrol group; similarly, the histopathological results corroborated these biochemical finding where the aortic specimen of the stem cells-treated group showed less foam cells than the resveratrol group. Qiao et al. (6) reported that the osteopetrotic (op/op) mice, which lack M-CSF due to a structural gene mutation, have a significant reduction in atherogenesis. They attributed their results to decreased circulating monocytes, reduced tissue macrophages, or diminished arterial M-CSF.

Stem cell treatment down-regulated the incident increment in the levels of VCAM and ICAM adhesion molecules in the atherosclerotic group Figure 2. Adhesion molecules are another pivotal factor mediating the adhesion of monocytes to the endothelial surface, followed by migration into the intima and differentiation into macrophages (30), whereas most of the latter transform into foam cells and result in the formation and release of free radicals and inflammatory cytokines into the circulation. Indeed, the histopathology of the atherosclerotic group showed abundant formation of foam cells in synchronization with the substantial increase in the levels of oxidative and inflammatory markers COX-2, iNOS, and TNF-α (Figure 3).

Studies in humans and experimental animals have found that an increased expression of those markers is associated with an increased intimal leucocyte accumulation and that there is an abundance of adhesion molecules on arterial sites prone to the development of atherosclerotic lesions (31). In addition to the decrease in the levels of adhesion molecules, the stem cells-treated rats showed a substantial down-regulation in both the oxidative stress and the inflammatory state, remarkably more than the treatment with resveratrol. Studies on stem cell repair of damage/lost endothelial cells in vein grafts and wire-injured arteries (32) in mice have found that genetic deficiencies of adhesion molecules are associated with significantly delayed atherosclerosis (31). Some studies indicate that macrophages involved in the process of atherosclerosis up-regulate protein and mRNA levels of iNOS and COX-2 (33). The induction of iNOS consistently occurs in atherosclerotic vessels of humans (34). However, studies have shown that SMCs as well as several other cell types respond to proinflammatory cytokines by transcribing the same iNOS gene as the macrophage (35).

Yan and Hansson (36) found that iNOS is rapidly induced in SMCs *in vivo* during the formation of the neointima. They documented that the high production of ROS by intimal SMCs is due to overexpression of iNOS. The atherosclerotic rats showed a significant increase in the levels of COX-2. The stem cells treatment restored COX-2 to normal levels while resveratrol treatment decreased them. COX-2 is expressed by endothelial cells, monocyte/macrophages, and vascular SMCs in atherosclerotic lesions of humans and murine models (37), which results in the formation and release of free radicals and inflammatory cytokines into the circulation (38); the COX inhibitor aspirin is shown to reduce the progression of coronary atherosclerosis (39).

Stem cells treatment substantially decreased the expression level of TNF-α while proliferating SMCs have demonstrated high expression of TNF-α (40).

Furthermore, in agreement with previous results, the stem cell treatment ameliorated and restored the oxidative balance in the atherosclerotic group in a superior manner to that exerted by resveratrol as manifested by significant increases of GSH and TAC levels and catalase activity. Stem cell treatment also caused a synchronized decrease in the percentage of DNA fragmentation.

## Conclusion

In conclusion, stem cells attenuated the experimentally induced atherosclerosis, shown by its remarkable capability in replacing one of the main contributors to the pathogenesis of arteriosclerosis, which is the neointimal SMCs. This was reflected by decreased protein levels of adhesion molecules and the gene expression of oxidative and inflammatory markers. Indeed, there was restoration in oxidative balance as well as the histopathological pattern in atherosclerotic rats.
